# Creating a Global Dialogue on Infectious Disease Surveillance: Connecting Organizations for Regional Disease Surveillance (CORDS)

**DOI:** 10.3402/ehtj.v6i0.19912

**Published:** 2013-01-25

**Authors:** Louise S. Gresham, Mark S. Smolinski, Rapeepong Suphanchaimat, Ann Marie Kimball, Suwit Wibulpolprasert

**Affiliations:** 1 Fondation Merieux USA, Inc., United States; 2 Skoll Global Threats Fund, United States; 3 International Health Policy Program, Ministry of Public Health, Thailand; 4 The Bill and Melinda Gates Foundation, United States; 5 Office of the Permanent Secretary, Ministry of Public Health, Thailand

**Keywords:** regional infectious disease surveillance network, global health security, network-of-networks, IHR implementation, WHO geopolitical structure

## Abstract

Connecting Organizations for Regional Disease Surveillance (CORDS) is an international non-governmental organization focused on information exchange between disease surveillance networks in different areas of the world. By linking regional disease surveillance networks, CORDS builds a trust-based social fabric of experts who share best practices, surveillance tools and strategies, training courses, and innovations. CORDS exemplifies the shifting patterns of international collaboration needed to prevent, detect, and counter all types of biological dangers – not just naturally occurring infectious diseases, but also terrorist threats. Representing a network-of-networks approach, the mission of CORDS is to link regional disease surveillance networks to improve global capacity to respond to infectious diseases. CORDS is an informal governance cooperative with six founding regional disease surveillance networks, with plans to expand; it works in complement and cooperatively with the World Health Organization (WHO), the World Organization for Animal Health (OIE), and the Food and Animal Organization of the United Nations (FAO). As described in detail elsewhere in this special issue of *Emerging Health Threats*, each regional network is an alliance of a small number of neighboring countries working across national borders to tackle emerging infectious diseases that require unified regional efforts. Here we describe the history, culture and commitment of CORDS; and the novel and necessary role that CORDS serves in the existing international infectious disease surveillance framework.

## Introduction

In the past few decades, one or two new infectious disease threats have emerged every year somewhere on the planet. The vast majority of new human infectious disease threats are zoonotic, meaning that they originate in animals. As we develop more land, mine more of the earth's resources, and hunt and raise more animals for food, we increase our exposure to animal pathogens that have the potential to “jump” the species barrier. Because both people and products are able to transit the globe during the incubation period for many infectious diseases, index cases often occur continents away from where outbreaks originate. Whether it be SARS in travelers (2003), influenza H1N1 in passengers (2009), Nipah virus in exported pigs (1998, 1999), HIV in shipped contaminated Factor VIII (1983), or *E. coli* in foodstuffs (ongoing), the geographic expanse of infectious diseases is truly boundless. Thus, populations across the globe are at risk of newly arriving infections that may be unfamiliar, difficult to diagnose, and challenging to treat and control (1–3).

At the same time, the increasingly competitive global marketplace has created new trading communities that are more economically integrated than in the past. New regional and cross border trade agreements have contributed to this shift in the trade landscape. The shift is driving many economies to begin to orchestrate their surveillance efforts, especially against infectious disease, with their neighboring trading and travel partners. Countries across the world are self-assembling into regional surveillance networks that do not necessarily operate within the confines of the older geo-political regional boundaries set forth by country membership in the World Health Organization (WHO) (4–6).

This article highlights the work being done by Connecting Organizations for Regional Disease Surveillance (CORDS). CORDS is an international non-governmental organization that links six of these self-assembling regional infectious disease surveillance networks (7–8). Each network is itself an alliance of a small number of neighboring countries working across national borders – sometimes borders in conflict – to tackle infectious disease threats that require unified regional efforts. By linking the networks not only with each other but also with WHO, the World Organisation for Animal Health (OIE), the Food and Agriculture Organization of the United Nations (FAO), and other agencies and institutions involved with disease surveillance, CORDS exemplifies the type of combined vertical plus horizontal international collaboration that will be needed to prevent, detect, and respond to the shifting spectrum of infectious disease threats that are an unfortunate reality in today's “global” world (9–10).

As elaborated throughout this issue, CORDS members regularly collaborate to build national, regional and global capacity while at the same time responding to infectious disease emergencies as they occur. The bonds that are forming within and among the CORDS regional surveillance networks are nurturing not just a cross-border approach to disease surveillance, but also a cross-disciplinary One Health approach. During times of emergency, the personal relationships being nurtured by CORDS and its member networks help to ensure that the joint outbreak investigations and controls are carried out in a timely manner; that the necessary diagnostics and potentially life-saving vaccines and drugs are shared where they are needed; that biological specimens are available for regional laboratory testing; and that appropriate regional travel restriction or other control measures are implemented when the situation demands.

Although this article highlights CORDS and its member networks, many other economies, government agencies, and informal groups have assembled in recent years to collectively combat infectious diseases (11–13). Examples include the West African Health Organisation (WAHO), the Pacific Public Health Surveillance Network (PPHSN), the Association of Southeast Asian Nations (ASEAN) Plus Three (China, Japan, Korea) Field Epidemiology Training Network (ASEAN+3 FETN), the Asia-Pacific Economic Cooperation (APEC) and APEC Emerging Infections Network (APEC EINet); the Caribbean Epidemiology Centre (CAREC); ProMED-mail; and the Global Outbreak Alert and Response Network (GOARN). See Ref. 11 in this issue for a discussion of WAHO, PPHSN, and ASEAN+3 FETN.

## History, Culture, and Commitment of CORDS

In 2007, the Rockefeller Foundation and Nuclear Threat Initiative (NTI) convened infectious disease surveillance representatives and other experts from across the world to share best practices and lessons learned in disease surveillance. Attendees of the meeting, which was held at the Rockefeller Foundation Bellagio Conference Center, Italy, were asked to recommend actions to advance the global capacity for public health surveillance and reduce the threat of infectious diseases, with a focus on the needs of developing countries. The resulting Bellagio Call for Action addressed three “vital concerns”: (i) the need to build surveillance capacity, especially human and laboratory capacity, but also cross-border collaborative capacity; (ii) the need to develop and employ appropriate information and data-sharing technology to facilitate timely communication during times of emergency; and (iii) the need for a flexible approach to governance among the growing number of regional infectious disease surveillance networks that are self-assembling worldwide (14).

At the same time, regional disease surveillance networks themselves were recognizing a shared incentive to improve early detection and outbreak investigation and response. Driven by that incentive and with the support and partnership of NTI, the Rockefeller Foundation, Peter G. Peterson Foundation, the Fondation Mérieux, and the Skoll Global Threats Fund, the leaders of six existing regional disease surveillance networks founded CORDS. The six networks, all of which are described in detail elsewhere in this issue (see also Table 1) are: Mekong Basin Disease Surveillance Network (MBDS) (15), East African Integrated Disease Surveillance Network (EAIDSNet) (16), South Eastern European Health Network (SEEHN) (17), Middle East Consortium on Infectious Disease Surveillance (MECIDS) (18), Asian Partnership on Emerging Infectious Disease Research (APEIR) (19), and Southern African Centre for Infectious Disease Surveillance (SACIDS) (20). During CORDS's early years, NTI served the role of interim secretariat; Fondation Mérieux provided a home in Annecy, France, for convening CORDS. CORDS was formally created as a non-governmental organization in Lyon, France, in 2012.

**Table 1:** Connecting Organizations for Regional Disease Surveillance (CORDS) Founding Networks

Name	Member Countries	Description
Mekong Basin Disease Surveillance Network (MBDS)	Cambodia, China (Yunnan and Guangxi Provinces), Laos PDR, Myanmar, Thailand, Vietnam	MBDS was established in 1999 to strengthen national and Mekong regional capabilities in disease surveillance and response to outbreaks of priority diseases in order that they can be effectively controlled. MBDS is governed by memoranda of understanding between the ministers of member countries and an executive board, with activities coordinated by a secretariat and country coordinators. For more information, visit www.mbdsoffice.com.
East African Integrated Disease Surveillance Network (EAIDSNet)	Burundi, Kenya, Rwanda, Tanzania, Uganda	EAIDSNet was established in 2001 to enhance cross-country and cross-institutional collaboration on disease control, to improve the quality of data on communicable disease and the flow and sharing of information, and to improve the health of the East African population. EAIDSNet is a health sector institution of the East African Community. For more information, visit www.eac.int/eaidsnet.
South-eastern Europe Health Network (SEEHN)	Albania, Bosnia and Herzegovina, Bulgaria, Croatia, Macedonia, Moldova, Montenegro, Romania, Serbia	SEEHN was founded in 2001 to coordinate and help with the implementation and evaluation of health policy and services among its regional members. The network is supported by a secretariat run jointly by the council of Europe and the WHO Regional Office for Europe. For more information, visit www.euro.who.int/en/what-we-do/health-topics/Health-systems/public-health-services/south-eastern-europe-health-network-seehn.
Middle East Consortium on Infectious Disease Surveillance (MECIDS)	Israel, Jordan, Palestinian Authority	MECIDS was established in 2003 to improve the ability of member nations to detect and respond to infectious disease threats through integrated surveillance systems and joint epidemiological and laboratory training. It is governed by an executive board guided by a set of standing operating procedures and associated protocols with activities coordinated by an international secretariat. For more information, visit www.mecidsnetwork.org.
Asian Partnership on Emerging Infectious Diseases Research (APEIR)	Cambodia, China, Indonesia, Vietnam, Thailand	APEIR was founded in 2006 by joining research efforts among different institutions in the most severely affected Asian countries to fight avian influenza in the region. It was initially named APAIR and further changed to APEIR when it expanded its interests to cover other emerging infectious diseases. Canada's International Development Research Centre (IDRC) is initially supporting the partnership. Key members include key multi-sectoral institutes in Cambodia, China, Indonesia, Thailand, and Vietnam. Other international agencies also supporting the alliance include AusAIDs, the Rockefeller Foundation, and WHO. For more information, visit www.apeiresearch.net/main.php.
Southern African Centre for Infectious Disease Surveillance (SACIDS)	Democratic Republic of Congo, Mozambique, South Africa, Tanzania, Zambia	SACIDS is a consortium of Southern African medical and veterinary, academic and research institutions in the animal, human and agricultural sectors. SACIDS was established in 2009 and is governed by two deputy directors, one for the human and the other for the animal health sector; at the national level, the coordinator is assisted by a deputy from the opposite sector with activities guided by a secretariat located in Tanzania. For more information, visit www.sacids.org.

Representing a network-of-networks approach, CORDS enables networks to interact not only with each other, but also with the WHO, OIE, FAO, and other surveillance partners. CORDS also partners with other public and private sector actors who share common health security goals (6, 7, 21, 22); and with individual professionals.

CORDS operates as a community of practice: a learning partnership among people who share a common concern, in this case improving infectious disease surveillance capacity, and who come together regularly to learn how to do it better (23). CORDS networks regularly meet to exchange information and innovations (e.g., new data-sharing tools); participate in training courses and learn through case studies; and jointly build surveillance capacity. By providing a central forum for peers from different parts of the world to share expertise and best practices and, over time, nurture trust, CORDS fosters the development of professional collaborations and provides regular opportunities for joint learning and technical exchanges. CORDS strengthens the dialogue not just among public health, veterinary, and wildlife professionals from different regions of the world, but also between those professionals and WHO, OIE, and FAO.

The vision of CORDS is “*a world united against infectious diseases*.” Its mission is “*to link regional disease surveillance networks and improve global capacity to respond to infectious diseases*.” CORDS has four strategic objectives:

1. Improving Capacity: CORDS facilitates the sharing between networks of case studies, technical expertise, data, best practices, and resources to help networks and their member countries develop new skills and build operational partnerships across regions.
2. Advancing One Health: CORDS seeks to modernize disease surveillance by improving coordination between animal, human and environmental sectors at national, regional and international levels.
3. Promoting Innovation: CORDS serves as a venue for networks to share their innovative ideas and approaches to disease surveillance, and it also provides an organized platform for co-development of new technologies and innovations within and across regions.
4. Building Sustainable Networks: CORDS strengthens multi-country disease surveillance networks and facilitates the creation of sustainable new networks in areas of high disease risk by providing educational materials, success stories, progress reports, and other information to networks which they can use with their respective ministries to demonstrate the value of multi-country networks.

While CORDS is still early in its development, already its member networks have demonstrated that even in parts of the world historically (e.g., Southeast Asia) or currently rife with conflict (e.g., Middle East), public health and veterinary experts and officials from neighboring countries can come together in emergency situations and successfully coordinate efforts to prevent the spread of infectious disease (4–6, 24). The key to success is trust. Multi-country disease surveillance networks are successful only when individual experts from across countries and regions develop trust-based relationships that support the comfortable and timely exchange of views and information. For example, MBDS is a network of trust-based social relationships that have developed over time and did not exist thirteen years ago. As the network matured and as disease surveillance and control epidemiologists and other professionals from neighboring countries routinely worked together on joint surveillance goals, the sharing of data, tools, and innovative ideas and approaches increased substantially. CORDS is committed to nurturing a trust-based culture that encourages the secure and timely sharing of information and best practices between disease surveillance experts from across its member networks.

The operational philosophy of CORDS is to be small, nimble, and supportive of member networks. In accord with the trust-based and collaborative culture of CORDS, the CORDS Executive Board operates on consensus when it establishes the objectives, policies, and plans of action for the organization. CORDS rotates leadership such that all involved networks will have the opportunity for one of their representatives to serve as Chair of the Executive Board (EB).

## Avenues for Engagement between CORDS and the Existing International Disease Surveillance Framework

Among the most obvious benefits of CORDS membership is that participating regional surveillance networks – and the countries linked by those networks – are improving national capacities in compliance with the revised International Health Regulations (IHR). The IHR provides a framework for improved international public health security by strengthening global surveillance, improving communication between WHO and member states, and setting ground rules to address national public health emergencies of international concern (25). It is a set of legally binding requirements agreed upon by the 194 member states to help structure a world that is on alert and prepared to respond to the threat of infectious epidemics. Many public health experts, including WHO leadership, have called for additional strategies to complement WHO efforts to build the mandated national capacities for compliance (5, 26–28). Indeed, the IHR itself includes provision for member states to seek technical assistance from WHO (29). However, WHO has limited funding and capacity to help countries meet capacity levels to detect and respond to cross-border threats. CORDS complements WHO efforts by helping regional networks to collectively built mandated core surveillance capacities through the sharing of information and standard protocols and the channeling of resources. WHO has been participating in CORDS activities and collaborating closely with the regional networks. WHO representatives regularly attend CORDS meetings; CORDS has held joint One Health meetings with WHO; and WHO has worked with both CORDS and its regional network members to build laboratory capacity.

CORDS fosters the sharing of information with other global infectious disease surveillance partners in addition to WHO, including OIE and FAO. Both are observers to the CORDS Executive Board. These collaborations are important to improving coordination between public health, veterinary, and wildlife surveillance and achieving the One Health strategic objective of CORDS.

Like its network members, CORDS cultivates trust-based relationships that enable neighboring countries to communicate and interact with each other during times of crisis more quickly and nimbly than is sometimes possible within the vertical and geopolitical structure of WHO. The vertical bureaucracy of WHO and member states dictates that permission from a top administrative unit is usually required to allow the sharing of information. The geopolitical structure of WHO is such that a number of neighboring countries are in different regions of WHO, which makes it difficult to communicate essential outbreak information via WHO channels in a timely manner. For example, Myanmar and Thailand are members of the South East Asia Region of WHO, while their neighbors China, Laos PDR, Cambodia, and Vietnam are members of the Western Pacific Region. Instead of relying solely on formal WHO communication channels during emergencies, neighboring countries are often able to launch more effective responses when they communicate directly with each other while at the same time communicating with WHO. The informal reporting structures of the regional disease surveillance networks featured here facilitate real-time information exchange and rapid and effective joint outbreak investigations. Moreover, CORDS networks also extend across non-member states.

The H5N1 pandemic threat in 2007 was a good example of informal communication facilitating a joint outbreak investigation between two countries in different WHO regions (15). The Thai team informed the Laos team immediately after the index case, in a Laotian girl, was diagnosed in Nong Khai province in Thailand, opposite the capital of Laos PDR, Vientiane. The Laos team immediately crossed the border to visit the girl, collect specimens, and start an investigation. The next day experts from the two countries conducted a joint outbreak investigation in the village where the girl resided, while at the same time reporting to WHO. This timely joint response was based on trust-based collegial relationships, without any requirement for permission from top leaders.

The trust-based social fabric being cultivated by CORDS and its member networks also helps to tackle the challenge of disease under-reporting, as well as wrongful accusations about neighboring WHO Member States. WHO is still limited in how it can intervene when a country does not report a disease threat (30).

## Value of CORDS

CORDS cultivates networks of professionals who have the collective strength to translate information into near-real-time action during emergency situations. The value of CORDS ranges from the potential (e.g., knowledge capital, social capital, learning capital) to the applied (e.g., changes in practice based on CORDS interactions) to the realized (improved performance). Text Box 1 outlines examples of the value of CORDS.

***Text Box 1*. The value of CORDS**

- **Weaving a strong global infectious disease surveillance fabric**. CORDS creates a social network for sharing information and documents, learning from experiences and common challenges, creating knowledge, stimulating change, and shaping new professional opportunities. The collective learning nurtured by CORDS turns short-term problem-solving into a long-term cumulative resource of approaches and solutions to infectious disease surveillance challenges; and creates horizontal channels of communication (e.g., network-to-network, country-to-country, network-to-private sector partner) that complement existing vertical structures (e.g., official WHO and Member State bureaucracies). Through these activities, CORDS weaves horizontal and vertical threads of communication and collaboration into a strong global infectious disease surveillance fabric. Examples of the horizontal collaboration being nurtured by CORDS include the spontaneity of the relationships among MBDS members being instrumental in forming the ASEAN Plus Three Centre for Emerging Infectious Disease, where 6 of the 13 members are MBDS members (15). Examples of vertical collaboration include WHO, OIE, and UN System Influenza Coordination (UNSIC) work with MBDS to develop scenarios and plan and carry out a series of pandemic preparedness tabletop exercises (19); WHO and MECIDS collaborating on IHR implementation strategies (18); and WHO assistance with the SEEHN regional assessment of national pandemic preparedness (17).
- **Contributing to global participatory policy reform**. CORDS plays a leadership role for policy reform on global issues (e.g., SACIDS advocacy for One Health policy [20], APEIR advocacy for research-based policy change [19]), draws additional policy attention to the concerns of regional networks, and creates a platform where national level action can be complemented by international level action.
- **Generating and managing knowledge**. CORDS generates and manages knowledge not only by carving new channels of communication so that surveillance data can be shared by individuals in a more timely manner than would otherwise be possible, but also by formulating and disseminating new surveillance norms and standards. CORDS also encourages the quick adoption of innovative technologies and innovations, such as point-of-care and rapid diagnostics (e.g., the joint SACIDS-EAIDSNet exploration of mobile technologies for disease surveillance in remote and cross-border areas [16]).

## Conclusion

The collaborative capacity to immediately detect, respond, and effectively control the occurrence of infectious diseases and prevent them from becoming pandemics is of utmost importance. While the IHR provides an official platform for infectious disease surveillance experts to communicate essential information, the vertical structure of WHO and the outdated geopolitical boundaries of the WHO regions create obstacles for infectious disease surveillance practitioners and other professionals to communicate in a timely manner during crises. Ensuring that a robust response to global infectious disease threats is present anywhere and everywhere at all times requires combining formal WHO and other surveillance mechanisms with the nimble nature of the regional networks linked by CORDS and of CORDS itself. The horizontal, semi-formal, trust-based relationships among regional disease surveillance networks being cultivated by CORDS interweave with the more formal, vertical relationships between Member States and WHO, OIE and FAO to form a global disease surveillance fabric that promotes more effective actions than would otherwise be possible. By pursuing a common vision where disease no longer threatens the security and prosperity of nations, CORDS is revitalizing international efforts against biological threats and helping to weave “A World United Against Infectious Diseases.”

## References

[1] Institute of Medicine [IOM] (2003). Microbial Threats to Health.

[2] Jones K (2008). Global trends in emerging infectious diseases. Nature.

[3] Institute of Medicine [IOM] (2006). The Impact of Globalization on Infectious Disease Emergence and Control: Exploring the Consequences and Opportunities.

[4] Leventhal A, Ralawi A, Belbiese A, Balicer RD (2006). Regional collaboration in the Middle East to deal with H5N1 avian flu. BMJ.

[5] Kimball AM, Moore M, French HM, Arima Y, Ungchusak K, Wibulpolprasert S (2008). Regional infections disease surveillance networks and their potential to facilitate the implementation of the International Health Regulations.

[6] Gresham L, Ramlawi A, Briski J, Richardson M, Taylor T (2009). Trust Across Borders: Responding to 2009 H1N1 Influenza in the Middle East. Biosecur Bioterror.

[7] Gresham L, Pray LA, Wibulpolprasert S, Trayner B (2011). Public Private Partnerships in trust-based public health social networking: Connecting Organizations for Regional Disease Surveillance (CORDS). J Commer Biotechnol.

[8] Gresham LS, Wibulpolprasert S, Leventhal A, Rweyemamu M (2011). Connecting Health Organizations for Regional Disease Surveillance (CHORDS): Transforming the International dialogue to Counter Biological Threats. Abstract No. 23. EcoHealth Journal.

[9] Khan AS, Fleischauer A, Casani J, Groseclose SL (2010). The next public health revolution: public health information fusion and social networks. Am J Public Health.

[10] The Royal Society Knowledge, Networks and Nations: Global Scientific Collaboration in the 21^st^ Century. http://royalsociety.org/uploadedFiles/Royal_Society_Content/Influencing_Policy/Reports/2011-03-28-Knowledge-networks-nations.pdf.

[11] Bond KC, Macfarlane S, Burke C, Ungchusak K, Wibulpolprasert S (2013). The evolution and expansion of regional disease surveillance networks and their role in mitigating the threat of infectious disease outbreaks. Emerging Health Threats.

[12] Castillo-Salgado C (2010). Trends and directions of global public health surveillance. Epidemiologic Reviews.

[13] Morse SS (2012). Public health surveillance and infectious disease detection. Biosecurity and Bioterrorism: Biodefense Strategy. Practice, and Science.

[14] Global Health and Security Initiative [GHSI] (2008). Bellagio call for action on public health surveillance networks: learning, trust, diplomacy, science and technology. Rockefeller Foundation Press Release.

[15] Phommasack B, Jiraphongsa C, Oo MK, Bond KC, Phaholyothin N, Suphanchaimat R (2013). Mekong Basin Disease Surveillance (MBDS): a trust-based network. Emerging Health Threats.

[16] Ope M, Sonoiya S, Kariuki J, Mboera LEG, Gandham RNV, Schneidman M, Kimura M (2013). Regional initiatives in support of surveillance in East Africa: the East Africa Integrated Disease Surveillance Network (EAIDSNet) experience. Emerging Health Threats.

[17] Bino S, Cavaljuga S, Kunchev A, Lausevic D, Kaic B, Pistol A (2013). Southeastern European Health Network (SEEHN) communicable diseases surveillance: a decade of building trust and collaboration. Emerging Health Threats.

[18] Leventhal A, Ramlawi A, Belbiesi A, Sheikh S, Haddadin A, Husseini S (2013). Enhanced surveillance for detection and management of infectious diseases: regional collaboration in the Middle East. Emerging Health Threats.

[19] Silkavute P, Tung DX, Jongudomsuk P (2013). Sustaining a regional emerging infectious disease research network: a trust-based approach. Emerging Health Threats.

[20] Rweyemamu MM, Mmbuji P, Karimuribo E, Paweska J, Kambarage D, Neves L (2013). The Southern African Centre for Infectious Disease Surveillance: a One Health consortium. Emerging Health Threats.

[21] Long WJ (2011). Pandemics and Peace. Public Health Cooperation in Zones of Conflict.

[22] Richter J (2004). Public-private partnerships for health: a trend with no alternatives?. Development.

[23] Wegner E (1999). Communities of Practice: Learning, Meaning, and Identity.

[24] Cohen D, Gargouri N, Ramlawi A (2010). Middle East subregional laboratory-based surveillance network on foodborne diseases established by Jordan, Israel, and the Palestinian Authority. Epidemiol Infect.

[25] Rodier G, Greenspan AL, Hughes JM, Heymann DL (2007). Global public health security. Emerging Infectious Diseases.

[26] Baker MG, Fidler DP (2006). Global public health surveillance under new International Health Regulations. Emerg Infect Dis.

[27] Sturvetant JL, Anema A, Brownstein JS (2007). The new International Health Regulations: considerations for global public health surveillance. Disaster Med Pub Health Prep.

[28] Institute of Medicine [IOM] (2010). Infectious Disease Movement in a Borderless World: Workshop Summary.

[29] World Health Organization (WHO) (2005). International Health Regulations, Second Edition. http://whqlibdoc.who.int/publications/2008/9789241580410_eng.pdf.

[30] Nuzzo J, Gronvall GK (2011). Global Health Security: Closing the Gaps in Responding to Infectious Disease Emergencies. Glob Health Gov.

